# *Araeoanasillus leptosomus* gen. et sp. nov., (Hemiptera, Cercopoidea, Sinoalidae?), a New Froghopper from Mid-Cretaceous Burmese Amber with Evidence of Its Possible Host Plant

**DOI:** 10.3390/life13040922

**Published:** 2023-03-31

**Authors:** George Poinar, Alex E. Brown

**Affiliations:** 1 Department of Integrative Biology, Oregon State University, Corvallis, OR 97331, USA; 2 629 Euclid Avenue, Berkeley, CA 94708, USA; amberbrownie@icloud.com

**Keywords:** Burmese amber, Cenomanian, froghopper, Sinoalidae, new genus

## Abstract

A new genus and species of froghopper, *Araeoanasillus leptosomus* gen. et sp. nov. (Hemiptera: Cercopoidea, Sinoalidae?), is described from mid-Cretaceous Burmese amber. The new genus possesses the following diagnostic characteristics: slender, medium size body (length, 7.0 mm) with head longer than wide, round eyes; antennae slender with eight antennomeres; pedicel very short, shorter than scape; pronotum with a length/width ratio of 2.4; metatibia with three spines, including one short spine near base and two adjacent, long, thick spines near apex; a single series of 16 thick apical teeth (comb) at metatibial apex; tegmen narrow with a length/width ratio of 3.2; tegmen with coastal area and stigmal cell punctate; CuP meeting base of CuA2; and MP branching at middle of wing. In hind wing, Cu vein forked once. A series of plant trichomes adjacent and attached to the specimen suggests that the froghopper’s host plant was a fern.

## 1. Introduction

The present study describes a new genus and species of froghopper (Hemiptera: Cercopoidea) in Burmese amber. Froghoppers comprise a large cosmopolitan group of diverse taxa extending back to the mid-Jurassic. The superfamily Cercopoidea presently contains five extant families (Aphrophoridae Amyot et Serville, 1843; Cercopidae Leach, 1815; Clastopteridae Dohrn, 1859; Epipygidae Hamilton, 2001; and Machaerotidae Stal, 1866) and three extinct families (Cercopionidae Hamilton, 1990; Procercopidae Handlirsch, 1906; and Sinoalidae Wang and Szwedo, 2012) [1,2].

With its diagnostic characters (slender, medium body size, head longer than wide, round eyes; metatibia with three spurs; a single crown of 16 thick spines at its tip; narrow tegmen with a length/width ratio of 3.2; tegmen with coastal area and stigmal cell punctate; CuP meeting base of CuA2 and MP branching at middle of wing; and hind wing Cu vein forked once), the present froghopper is tentatively placed in the family Sinoalidae.

**Figure 1:**
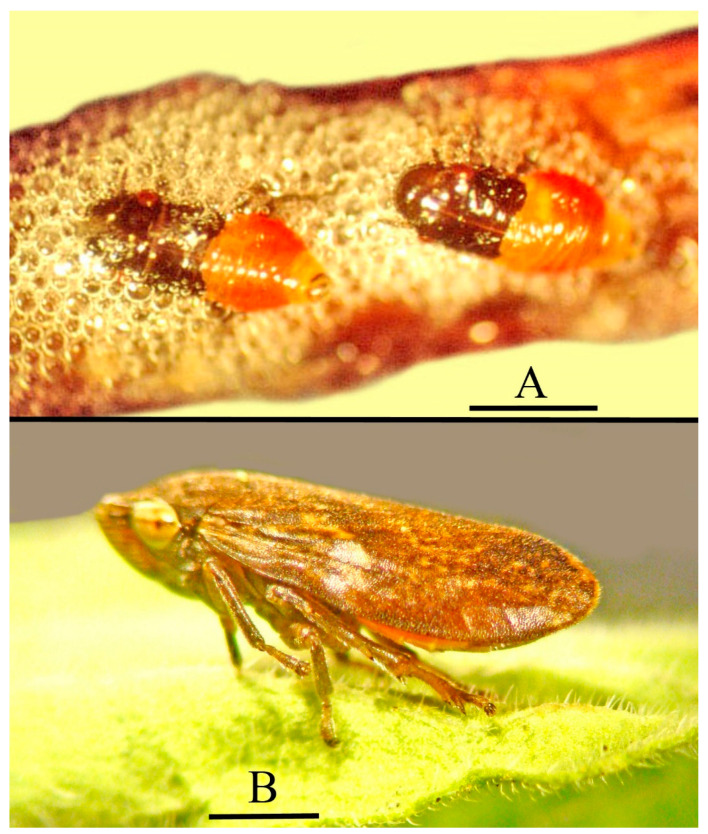
(**A**) Nymphs of *Aphrophora* sp. amidst bubbles on salal (*Gautheria shallon*) in Oregon. (Photo by G. Poinar, Jr.) Scale bar = 1.7 mm. (**B**) Adult *Aphrophora* sp. on Sand Dune Phacelia (*Phacelia argentea*) in Oregon. (Photo by G. Poinar, Jr.). Scale bar = 1.6 mm.

Extant members of the Cercopoidea have two behavioral patterns during their development. While the nymphs, which are called spittlebugs, produce froth and feed in a surrounding mass of spittle that masks their presence (Figure 1A), the flying adults, which are called froghoppers, lack this protection (Figure 1B).

While we know nothing about the biology of extinct froghoppers (food preferences, feeding habits, parasites, whether the nymphs were able to produce froth during their development), the present specimen provides us with clues to its possible plant host. Unique plant trichomes adjacent to and attached to the fossil suggest that the froghopper fed and likely oviposited on ferns.

## 2. Materials and Methods

The fossil froghopper originated from the Noije Bum 2001 Summit Site mine located southwest of Maingkhwan in Kachin State (26°20′ N, 96°36′ E) in Myanmar. Based on paleontological evidence, this site was dated to the Upper Albian of the Early-Mid Cretaceous [3], placing the age at 97 to 110 mya. A more recent study using U-Pb zircon dating determined the age to be 98.79 ± 0.62 Ma [4]. Nuclear magnetic resonance (NMR) spectra and the presence of araucaroid wood fibers in amber samples from the Noije Bum 2001 Summit Site indicate an araucarian tree source for the amber [5].

Observations and photographs were made with a Nikon SMZ-10 R stereoscopic microscope and Nikon Optiphot compound microscope with magnifications up to 800×. (Nikon Inc. 1300 Walt Whitman Road, Melville, NY), Helicon Focus Pro X64 (GT Vision Co, UK) was used to stack photos for a better depth of field. Tegminal venation and morphological terminologies are based on those used by Chen et al. [1].

## 3. Results

### Systematic Paleontology

Order **Hemiptera** Linnaeus, 1758

Suborder **Cicadomorpha** Evans, 1946

Superfamily **Cercopoidea** Leach, 1815

Family **Sinoalidae** Wang and Szwedo, 2012 (provisional placement)

Subfamily and Tribe unknown

Genus **Araeoanasillus** Poinar et Brown **gen. nov.** (Figure 1, Figure 2, Figure 3, Figure 4, Figure 5, Figure 6, Figure 7, Figure 8 and Figure 9)

Type species *Araeoanasillus leptosomus* Poinar et Brown **sp. nov.**

LSID for genus: 9D5ABA45-B3CC-4D43-95DE-03D035B46670

**Etymology**. The generic name is derived from the Greek “araeos” = thin and the Greek “anasillos” = bristling hair, in reference to the surrounding trichomes.

**Diagnosis**. Slender, medium size body (length, 7.0 mm) with head longer than wide, round eyes; antennae slender with eight antennomeres; pedicel very short, shorter than scape; pronotum with a length/width ratio of 2.4; metatibia with three spines, including one short spine near base and two adjacent, long, thick spines near apex; a single series of 16 thick apical teeth (comb) at metatibial apex; tegmen narrow with a length/width ratio of 3.2; tegmen with coastal area and stigmal cell punctate; CuP meeting base of CuA2; MP branching at middle of wing; and radial cell (C1) very narrow. 

**Comments**. *Araeoanasillus leptosomus* can be separated from the 14 previously described Burmese amber sinoalid species by having the CuP meeting the base of CuA2 in the fore wing. All previously described sinoalid species have CuP and CuA2 clearly meeting the peripheral margin separately [2]. The feature of the forewing CuP meeting the base of CuA2 is actually a basic character for members of the Procercopidae [2], which is why *Araeoanasillus* is only provisionally placed in the Sinoalidae.

***Araeoanasillus leptosomus*** Poinar et Brown **sp. nov.** (Figure 2, Figure 3, Figure 4, Figure 5, Figure 6, Figure 7, Figure 8, Figure 9, Figure 10 and Figure 11)

LSID for species: 054A-47A7-7967-4782-00DD3CA9E06D

**Etymology**: The specific epithet is derived from the Greek “leptos” = slender and the Greek “soma” = body in reference to the slender shape of the fossil.

**Type material**. Holotype female deposited in the Poinar amber collection (accession # B-He-41) maintained at Oregon State University.

**Type locality**. Hukawng Valley southwest of Maingkhwan in Kachin State (26°20′ N, 96°36′ E), Myanmar.

**LSID** for publication: 9BA4F407-D8FD-47D5-A71E-557O66BE0C-AC

Description

**Holotype** female. Entire body various shades of brown.

**Figure 3:**
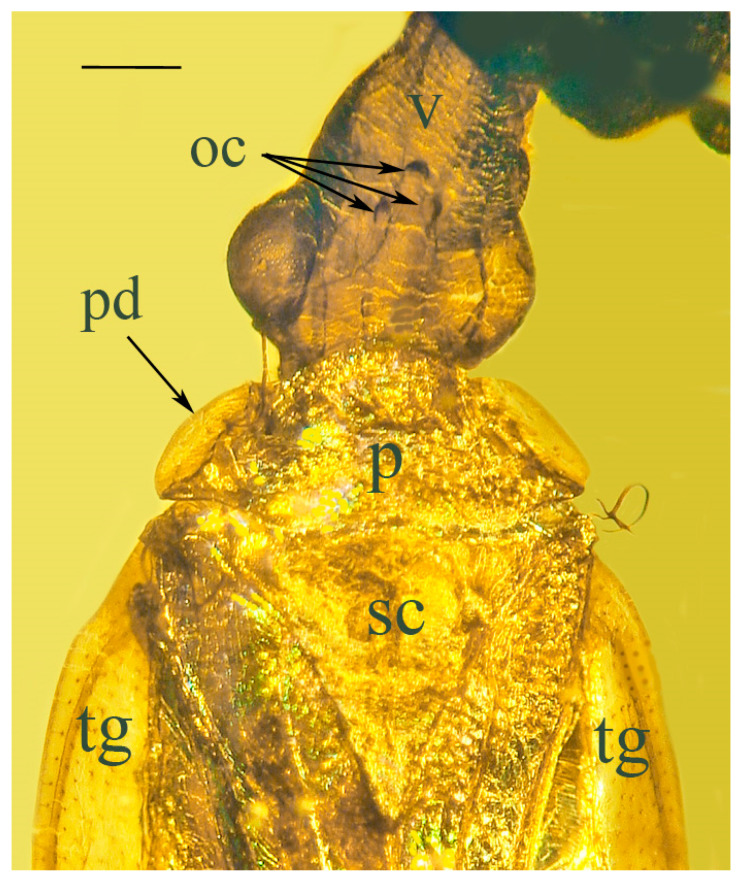
Dorsal view of head of holotype of *Araeoanasillus leptosomus* gen. et sp. n. in Burmese amber. oc = ocelli; pd = pronotal disc; p = pronotum; sc = scutellum; tg = tegmen; v = vertex. Scale bar = 430 µm.

Head (Figure 3): Longer than wide. Vertex declivous anteriorly, protruding well in front of eye level. Three ocelli, the two lateral positioned at level of anterior margin of compound eyes; the third ocellus positioned anterior to margin of compound eyes. Compound eyes large, globular, distinctly protruding from surface of head; and rostrum short, three-segmented, extending only to level of procoxae, with styles extended. Antenna located above eyes with long scape, short pedicel and six flagellomeres.

Thorax: Pronotum fairly short, with rounded anterior margin covering neck and reaching to base of compound eyes; lateral surfaces with flattened, angled pronotal discs; and posterior margin slightly curved medially. Mesonotum with faint median carina. Scutellum triangulate, not separated by transverse carina.

Legs: Metatibia with three spines, one short spine near base and two adjacent long thick spines near apex; a single series of 16 thick apical teeth (comb) at apex of metatibia; and three tarsomeres approximately equal in length, with a pair of long claws and arolium.

Wings: Tegmina transparent, exceeding length of body with L/W ratio = 3.0; top margin distinctly curved; basal margin straight; costal cell and claval area punctate; cell between costal margin and Pc + CP narrow; stem of MP forked at mid-wing level, reaching margin with two terminals (MP1 + 2 and MP3 + 4); stem CuA slightly curved at base; CuA1 curved mediad at base, reaching postclaval margin basad of posteroapical angle; branch CuA2 slightly curved near base; veinlet ir slightly distad of veinlet imp; and CuP meeting base of CuA2.

Hind wing membranous, apex arcuate; posterior margins entire; Cu vein forked basal to mid-wing; branch CuP single, straight; veins Pcu and A1 slightly curved; and anal lobe not observed.

**Figure 11:**
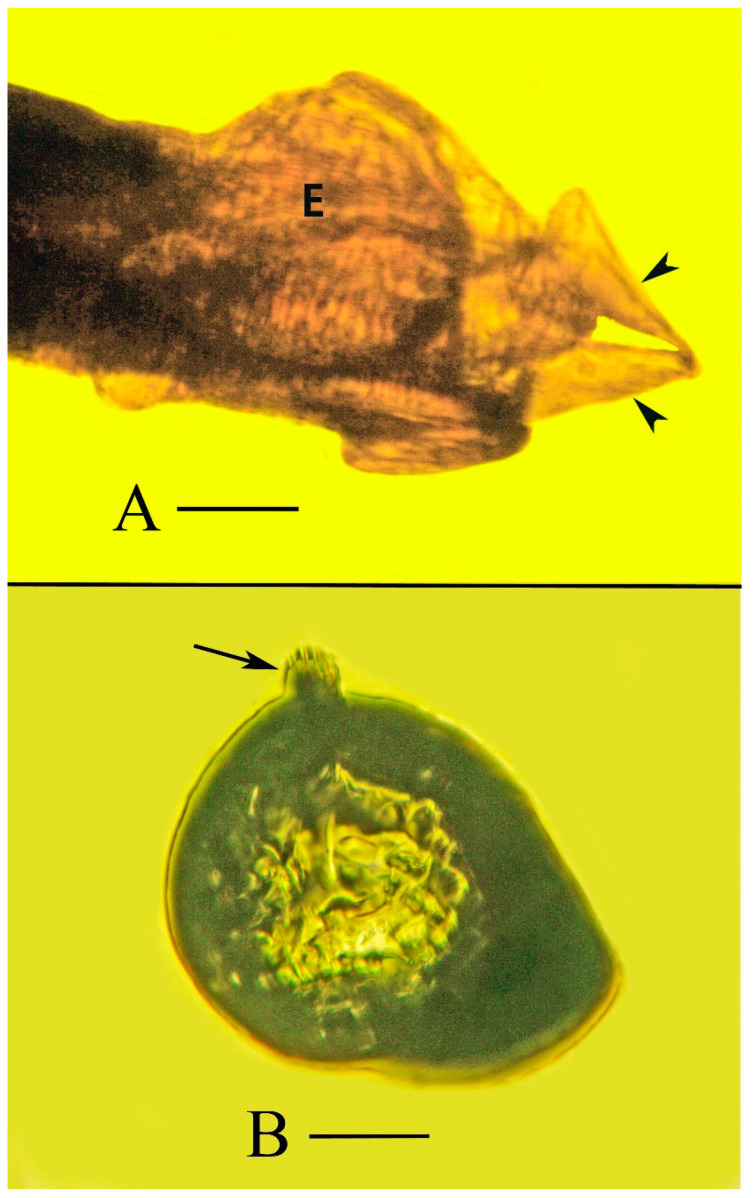
(**A**). Dorsal view of abdominal terminus of holotype of *Araeoanasillus leptosomus* gen. et sp. n. in Burmese amber showing a possible egg (E) ready to be deposited. Note valves of ovipositor (arrowheads). Scale bar = 86 µm. (**B**). Deposited tentative egg of *Araeoanasillus leptosomus* gen. et sp. n. showing possible developing embryo and respiratory horn (arrow). Note the structure is asymmetrical, possibly indicating stretching by growth or movement of a possible embryo. Scale bar = 67 µm.

Abdomen: With seven visible segments, narrow, outstretched, with short inner and outer ovipositor valves and terminal egg (see Figure 11A).

Measurements: Length body, 7.0 mm; width body 0.8 mm; body L/W ratio, 8.7; width head across eyes, 1.5 mm; length eye (lateral), 1.5 mm; width eye, 0.6 mm; length tip of head to pronotum, 1.6 mm; length scape, 49 µm; length pedicel, 21 µm; length flagellum, 224 µm; length exposed mandibular stylet, 630 µm; length exposed maxillary stylet, 770 µm; length pronotum, 1.9 mm; width pronotum, 0.8 mm; (L/W ratio, 2.4); length mesonotum, 1.4 mm; width mesonotum, 1.8 mm; length rostrum, 1.0 mm; length scape, 49 µm; length pedicel, 21 µm; length flagellum, 224 µm; length metatibia, 0.7 mm; length metafemur, 0.7 mm; length metatarsomere, 0.4 mm; length claws, 320 µm; length arolium, 213 µm; length tegmen, 10.6 mm; width tegmen, 3.5 mm; and tegmen L/W ratio, 3.0.

**Figure 12:**
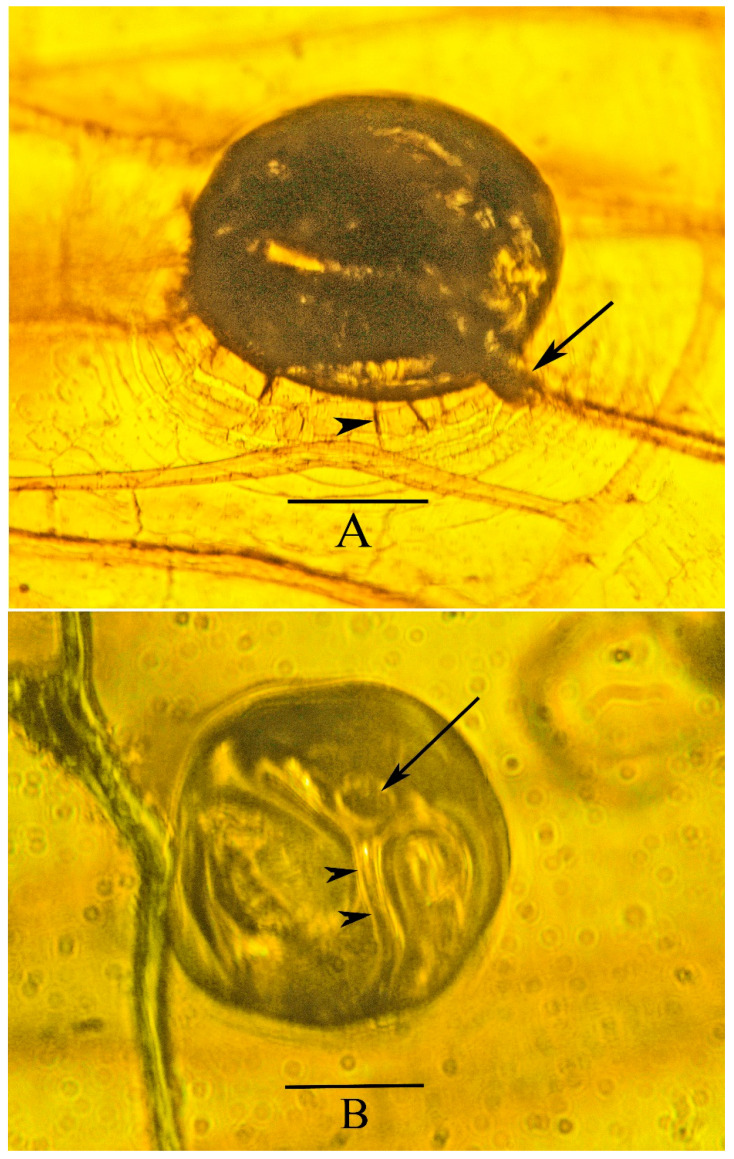
(**A**). Anterior end of a possible deposited egg of the holotype of *Araeoanasillus leptosomus* gen. et sp. n. in Burmese amber, with wing in background. Note possible respiratory horn (arrow) and one of several spines on chorion (arrowhead). Scale bar = 93 µm. (**B**). Possible deposited egg of *Araeoanasillus leptosomus* gen. et sp. n., with wing in background, showing possible developing embryo and respiratory horn (arrow). Note tube leading from respiratory horn to inside tentative egg (arrowheads). Scale bar = 80 µm.

At the end of the reproductive tract of *Araeoanasillus* is a circular structure that is interpreted to be an egg ready to be deposited (Figure 11A). Three additional structures are believed to be eggs that have already been deposited in the amber adjacent to the specimen (Figure 11B and Figure 12A,B). It is common for female insects to deposit eggs in their final moments after they fall in resin [6]. However, being dark and mostly circular in shape, these tentative “eggs” differ from the oval–elliptical, pale-colored eggs of extant froghoppers [7]. It is possible that members of extinct families of froghoppers had a variety of egg hues and shapes.

Insect eggs possess various modifications for air and/or water exchange. The interior chorion can harbor a thin layer that connects directly to the atmosphere by openings called aeropyles or plastrons. Other insect eggs, such as the circular structures near *Araeoanasillus*, possess a “respiratory process” or “respiratory horn” at the anterior end that allows air to enter the egg during embryonic development (Figure 11B and Figure 12A,B) [8,9]. In one of the tentative eggs depicted here (Figure 11B) is what appears to be a developing embryo.

## 4. Discussion

The diagnostic characters of *Araeoanasillus leptosomus* separate the genus from previously described Burmese amber froghopper fossils [2]. Features of the forewing venation place *Araeoanasillus* in a group of taxa are considered to have evolved either from “primitive” lineages related to Procercopidae or from a “pre-Procercopidae“ group [2,10]. Based on the available characters, the fossil has features of both the Procercopidae and the Sinoalidae.

Cercopoid lineages occur predominately on angiosperms today. However, in the early Mesozoic, when extinct froghoppers existed, there were no uncontested angiosperms [11,12,13,14]. The uniquely shaped trichomes found surrounding and attached to the abdomen and legs are significant in determining a possible plant host of *Araeoanasillus leptosomus*. These plant hairs are similar to fern trichomes and compare with those presented by Pérez-de la Fuente et al. [15], as well as the branched trichomes found adjacent to the Burmese amber fern, *Cladarastega burmanica* [16], and what Thompson and Martin [17] call “gnarled trichomes”. Some of the trichomes have attached glands, a phenomenon found in a number of plants [18]. It is likely that the host fern was growing at the base of, or on, one of the branches of a kauri tree (Araucariaceae) that was producing resin [5]. Since some trichomes are intricately associated with *A. leptosomus,* it is doubtful that they could have simply been blown or fallen on the froghopper since there is no other extraneous debris present.

While the adjacent beetle could have been entombed by chance, extant adults and larvae of Nosodendridae are considered to be predaceous and live in oozing wounds and slime fluxes of angiosperm and gymnosperm trees. If the habits of ancient members were similar to those of today, the beetle could have been feeding on the nymphs of *A*. *leptosomus* [19].

The most common hemipterans feeding on ferns today are aphids, which also date back to the Early Mesozoic [20,21]. In addition, no such trichomes have been found among the 31 angiosperm flowers that have been described from Burmese amber [13].

While some aphid lineages still maintain their preference for ferns [20], records of froghoppers on ferns are rare [22]; however, spittlebugs of *Philaenus spumarius* (L.) have been found on ferns in Bulgaria [23] and Poinar [20] noted these insects feeding on *Pteridium aquilinum* along the Oregon coast. However, over the eons, almost all cercopoid lineages have shifted to angiosperms.

*Araeoanasillus leptosomus* adds to the amazing diversity of mid-Cretaceous Cercopoidea [1,2,10,24,25] and provides evidence that the fossil was probably feeding on ferns.

## Figures and Tables

**Figure 2:**
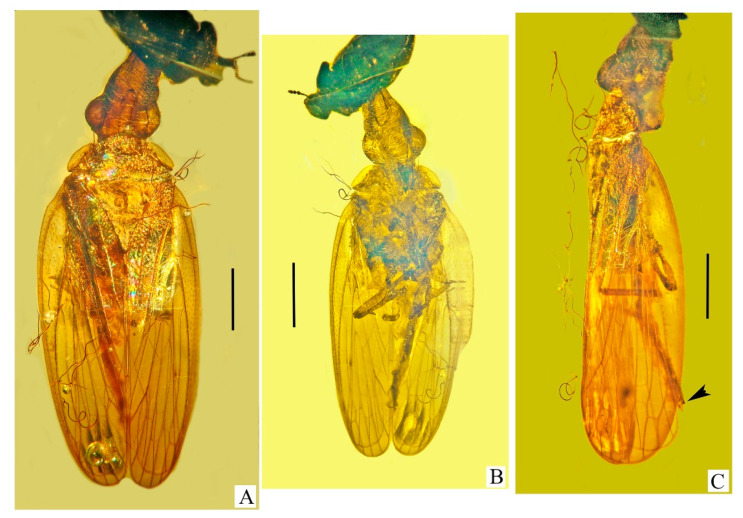
Holotype of *Araeoanasillus leptosomus* gen. et sp. n. in Burmese amber. (**A**)—dorsal view; (**B**)—ventral view; (**C**)—lateral view. Arrowhead shows tip of abdomen. Scale bar for (**A**,**C**) = 1.0 mm; for (**B**) = 1.3 mm.

**Figure 4:**
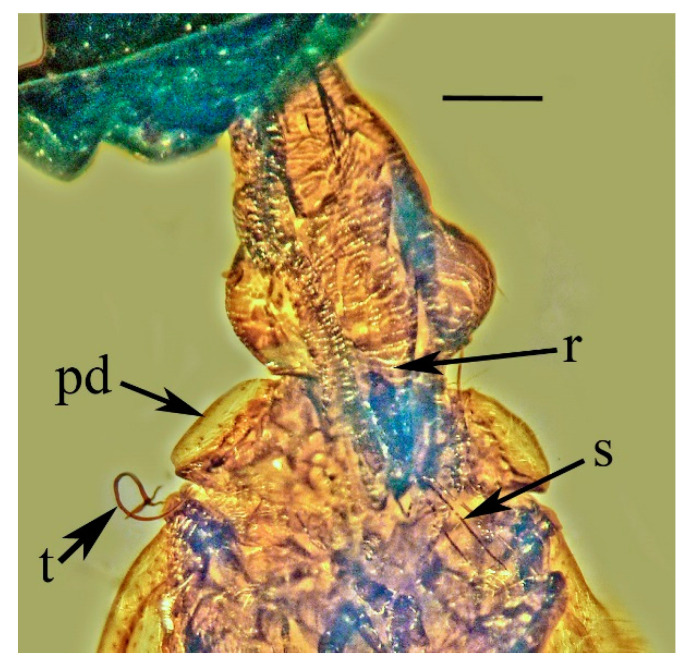
Ventral view of head of holotype of *Araeoanasillus leptosomus* gen. et sp. n. in Burmese amber. pd = pronotal disc; r = rostrum; s = style; t = trichome. Scale bar = 400 µm.

**Figure 5:**
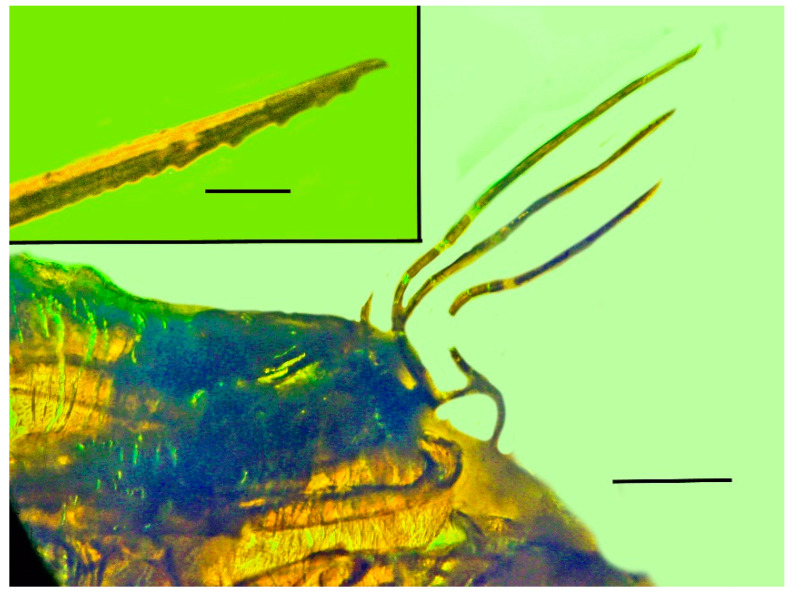
Three extended stylets emerging from the tip of the rostrum of the holotype of *Araeoanasillus leptosomus* gen. et sp. n. in Burmese amber. Scale bar = 180 µm. Insert shows serrations on mandibular stylet. Scale bar = 60 µm.

**Figure 6:**
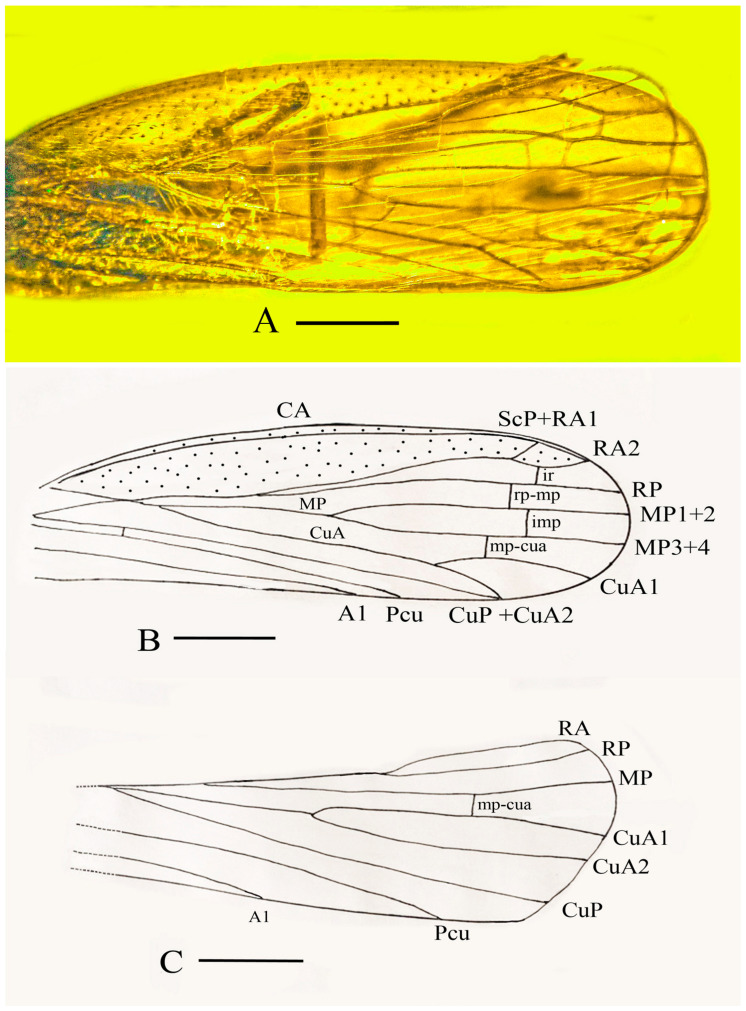
Holotype of *Araeoanasillus leptosomus* gen. et sp. n. in Burmese amber. (**A**)—Wings; (**B**)—Labeled forewing; (**C**)—Labeled hind wing. Scale bar for (**A**) =740 µm, for (**B**,**C**) = 0.8 mm.

**Figure 7:**
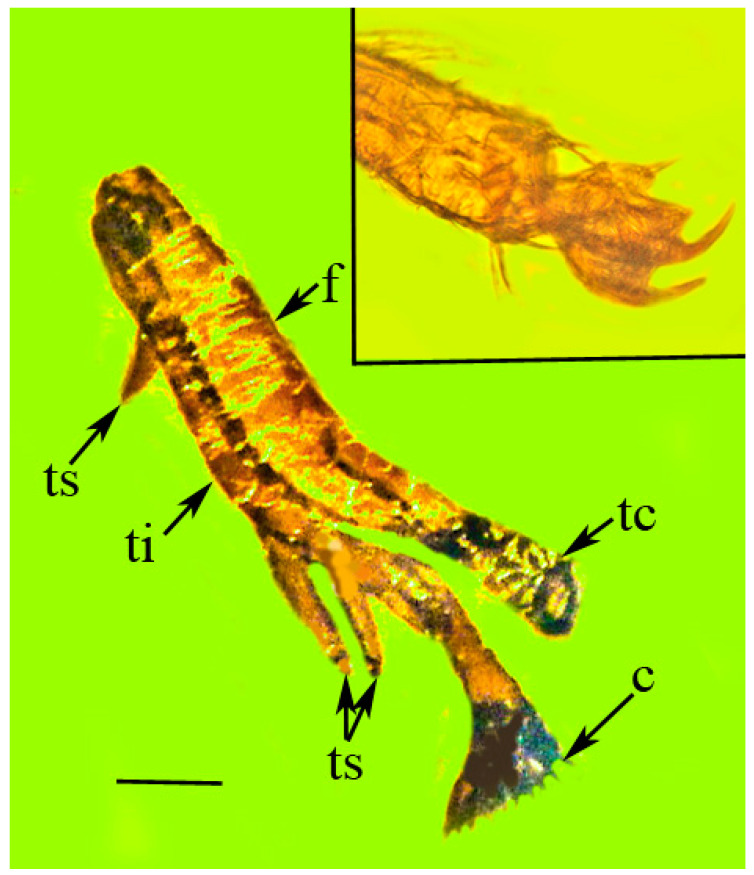
Hind leg of holotype of *Araeoanasillus leptosomus* gen. et sp. n. in Burmese amber. c = cluster of tibial teeth; f = femur; tc = coxa and trochanter; ti = tibia; ts = tibial spurs. Scale bar = 350 µm. Insert shows paired claws and arolium. Scale bar = 70 µm.

**Figure 8:**
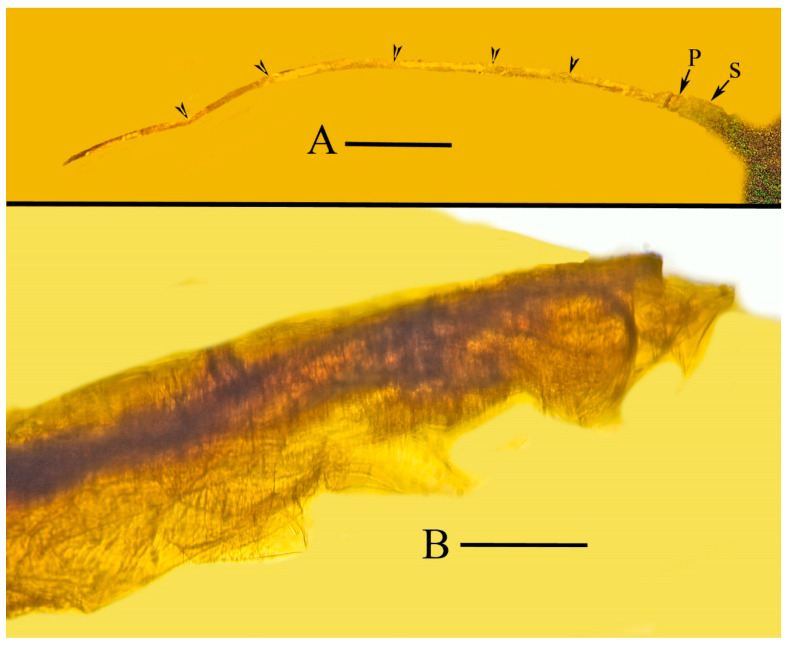
(**A**). Antenna of holotype of *Araeoanasillus leptosomus* gen. et sp. n. in Burmese amber. P = pedicel; S = scape. Arrowheads show divisions between segments. Scale bar = 36 µm. (**B**). Tip of abdomen (lateral) of *Araeoanasillus leptosomus* gen. et sp. n. in Burmese amber. Scale bar = 200 µm.

**Figure 9:**
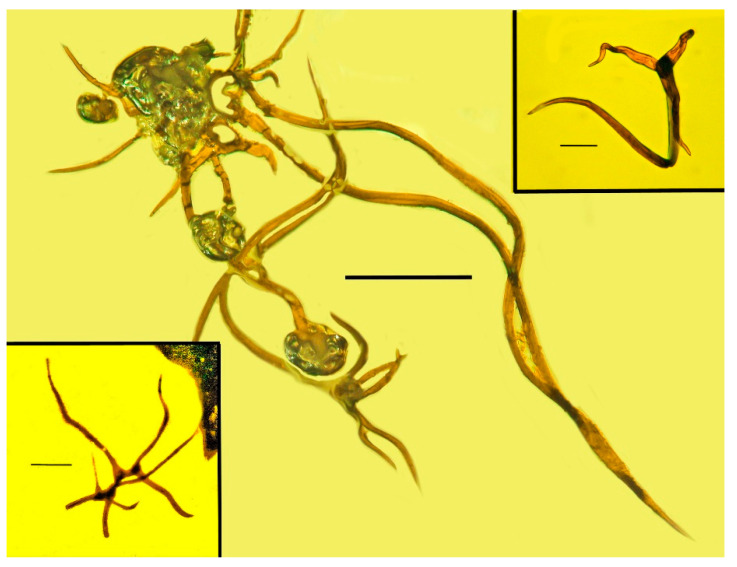
Glandular trichomes adjacent to holotype of *Araeoanasillus leptosomus* gen. et sp. n. in Burmese amber. Scale bar = 200 µm. Right upper insert shows non-glandular trichome adjacent to holotype. Scale bar = 56 µm. Left lower insert is a trichome from the Burmese amber fern, *Cladarastega burmanica*. Scale bar = 100 µm.

**Figure 10:**
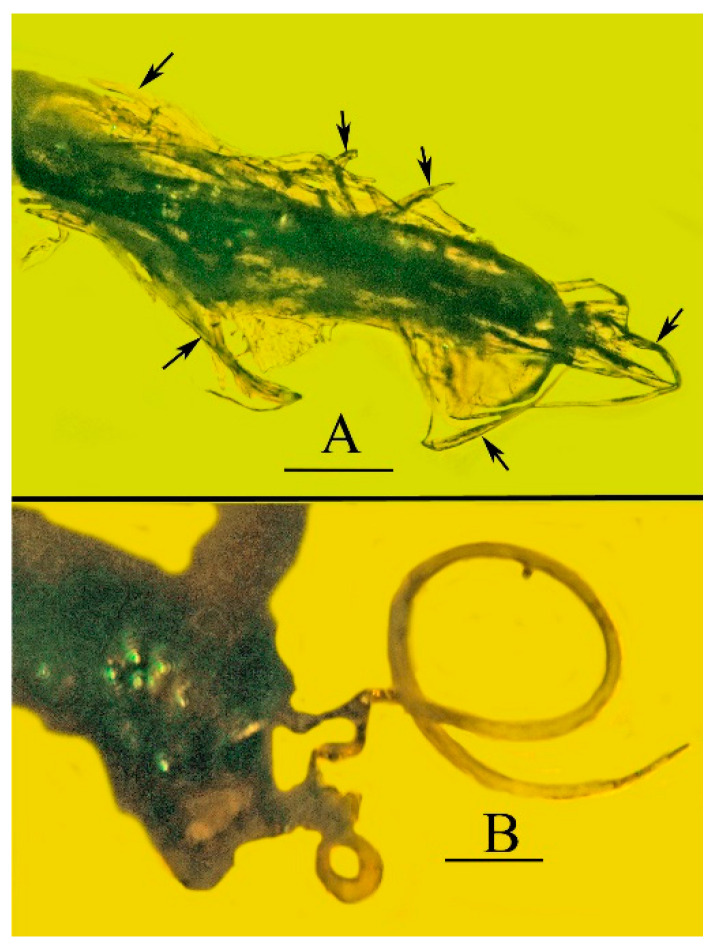
Trichomes attached to holotype of *Araeoanasillus leptosomus* gen. et sp. n. in Burmese amber. (**A**). Trichomes (arrows) adhering to tip of abdomen. Scale bar = 150 µm. (**B**). Trichome attached to hind leg. Scale bar = 105 µm.

## Data Availability

Data availability can be found in the references.
